# Procalcitonin in liver transplantation: are high levels due to donors or recipients?

**DOI:** 10.1186/cc6942

**Published:** 2008-07-04

**Authors:** Daniel Eyraud, Saïd Ben Ayed, Marie Laure Tanguy, Corinne Vézinet, Jean Michel Siksik, Maguy Bernard, Sylvia Fratéa, Marie Movschin, Jean-Christophe Vaillant, Pierre Coriat, Laurent Hannoun

**Affiliations:** 1 Département d'Anesthésie-Réanimation, Hôpital Pitié-Salpêtrière 43-47 Boulevard de l'Hôpital, 75013 Paris, France; 2 Laboratoire de Biochimie, Hôpital Pitié-Salpêtrière 43-47 Boulevard de l'Hôpital, 75013 Paris, France; 3 Unité de Recherche Clinique, département de Statistiques, Hôpital Pitié-Salpêtrière 43-47 Boulevard de l'Hôpital, 75013 Paris, France; 4 Service de chirurgie digestive et de transplantation hépatique, Hôpital Pitié-Salpêtrière 43-47 Boulevard de l'Hôpital, 75013 Paris, France

## Abstract

**Introduction:**

To date, a specific marker to evaluate and predict the clinical course or complication of the liver-transplanted patient is not available in clinical practice. Increased procalcitonin (PCT) levels have been found in infectious inflammation; poor organ perfusion and high PCT levels in the cardiac donor appeared to predict early graft failure. We evaluated PCT as a predictor of early graft dysfunction and postoperative complications.

**Methods:**

PCT serum concentrations were measured in samples collected before organ retrieval from 67 consecutive brain-dead donors and in corresponding recipients from day 0, before liver transplantation, up to day 7 after liver transplantation. The following parameters were recorded in donors: amount of vasopressive drug doses, cardiac arrest history 24 hours before retrieval, number of days in the intensive care unit, age of donor, and infection in donor, and the following parameters were recorded in recipients: cold and warm ischemia time, veno-venous bypass, transfusion amount during orthotopic liver transplantation (OLT), and occurrence of postoperative complication or hepatic dysfunction.

**Results:**

In the donor, the preoperative level of PCT was associated with cardiac arrest and high doses of catecholamines before organ retrieval. In the recipient, elevated PCT levels were observed early after OLT, with a peak at day 1 or 2 after OLT, then a decrease until day 7. A postoperative peak of PCT levels was associated neither with preoperative PCT levels in the donor or the recipients nor with hepatic post-OLT dysfunction or other postoperative complications, but with two donor parameters: infection and cardiac arrest.

**Conclusion:**

PCT level in the donor and early PCT peak in the recipient are not predictive of post-OLT hepatic dysfunction or other complications. Cardiac arrest and infection in the donor, but not PCT level in the donor, are associated with high post-OLT PCT levels in the recipient.

## Introduction

Procalcitonin (PCT) is a 116-amino acid precursor protein of calcitonin and, in 1992, was identified as a new diagnostic marker for various processes [1-3]. Normally, in healthy individuals, PCT serum concentrations are very low, often even below the detection limit of the presently used assay. The *in vivo *half-life of PCT is approximately 24 to 30 hours [2,4]. Elevated PCT levels are observed early after orthotopic liver transplantation (OLT) [5]. The origin of inflammatory synthesis-induced PCT has not been clarified yet: neuroendocrine cells of different organs (lung, intestinium, kidney, pancreas, adrenal gland, and more recently the liver) have been proposed as a major source of PCT production [1]. The main stimulus for PCT induction is probably a systemic challenge of the organism with bacterial endotoxin (bacterial lipopolysaccharides) [2]. Because the delay between the induction of PCT synthesis and the increase in serum level is short [3,4], the elevated level of PCT just after OLT [5,6] can be due to recipient causes or donor causes. Moreover, if the liver is a major source of PCT production, serum levels of PCT could vary could vary with a given factor, depending on the liver graft. The aim of this prospective study was, first, to clarify in a large cohort of consecutive patients whether PCT in the donor or early in the recipient could be predictive of hepatic dysfunction or complications of other causes. Second, we tried to identify parameters associated with an increase in PCT in donors and recipients.

## Materials and methods

After approval by the local ethics committee, all patients admitted for liver transplantation at our institution, Pitié Salpétrière Hospital (Assistance Publique-Hôpitaux de Paris), between July 2003 and March 2005 were prospectively included in the study. The ethical committee waived the need for informed consent because alicots were taken from routine samples. For each recipient, the following were recorded: age, gender, presence of severe portal hypertension, need for veno-venous bypass, number of blood cell transfusions, and PCT serum concentration before anhepatic phase and then 12 hours after reperfusion and daily during the first week after OLT. Postoperative clinical course was analyzed from main clinical data: hepatic dysfunction, pulmonary and renal failure, and overall complications. For each donor, the following were collected: full cadaveric, age, occurrence of cardiac arrest (CA) 24 hours before the retrieval of the organs, occurrence of infection (I), possibility of retrieving the heart with the liver, amount of catecholamine (epinephrine or norepinephrin) administered before organ retrieval, and number of days in the intensive care unit before retrieval. All organs were retrieved and flushed using the same procedure: Wisconsin solution for preservation and 4% human albumin solution for hepatic flush before graft reperfusion.

### Procalcitonin measurement

Blood samples were obtained for routine testing (biochemical parameters), and for each patient, serum aliquots were used for PCT determination. A blood sample from the donor was obtained after installation of the donor in the operating room. An investigator blinded to clinical data used a time-resolved amplified cryptate emission technology on a Kryptor analyser (Brahms Diagnostica GmbH, Berlin, Germany) to measure PCT in 100 μL of serum. The analytic sensitivity of the assay was 0.06 ng/mL and the detection threshold was 0.02 ng/mL; the normal range detected was from 0.1 to 0.5 ng/mL.

### Definitions

Graft dysfunction was defined as the occurrence of at least one of the following four criteria: the need for retransplantation (primary nonfunction, PNF), a rise in aminotransferases of above 2,000 UI/L [7], the need for plasma transfusion for hemorrhagic ascites in relation to factor V of less than 30% or poor discolored bile, and an increase in bilurubinemia without a retrospective need for retransplantation. Death was defined as death from any cause occurring during the hospital stay. Pulmonary complication was defined as continuation of mechanical ventilation for more than 48 hours or the need to replace mechanical ventilation the first week post-OLT. Acute renal failure was defined as plasma creatinemia of greater than 180 μmol/L and urine output of less than 0.5 mL/hour. Renal complication was defined as the need for dialysis after OLT or greater than 100% of creatinine levels compared with preoperative values. Postoperative complication was defined as hepatic dysfunction, infection, or pulmonary or renal complication. Infection was diagnosed if microbiological cultures obtained from the patients at possible sites of infection were positive (proven infection) or if clinical signs of infection were evident. Pneumonia was diagnosed if radiological signs of pneumonia (infiltration) on chest x-rays and at least one of the following two criteria was present: leukocytosis of greater than 12,000 × 10^9^/L or less than 4,000 × 10^9^/L or body temperature of greater than 38°C or less than 36°C. Severe portal hypertension in recipients was defined as hepatic venous portal gradient of greater than 20 mm Hg if the patient had a preoperative hepatic transjugular exploration or a decrease in portal output of less than 500 mL/minute, estimated with Doppler ultrasonography. Poor tolerance to vascular liver exclusion was defined as a macroscopic disturbance of bowel coloration or persistence of mean arterial pressure of less than 50 mm Hg and oxygen mixed venous saturation of less than 60% despite fluid loading.

### Clinical protocols

All patients were treated using our standard protocol for immunosuppression: cyclosporine A (trough residual concentration of 200 to 400 ng/mL at day 7) or FK 507 (trough concentration of 10 to 15 ng/mL at day 7), prednisolone, starting at 10 mg/kg of body weight on the day of transplantation and reduced to half doses each day to 0.3 mg/kg at day 7, and mycophenolate mofetil (1 g per day) from the day of the liver transplantation. Acute rejection was diagnosed based on clinical and biochemical data and liver biopsy if required. All recipients received broad-spectrum antibiotic treatment with piperacilline-tazobactam for 7 days and ciprofloxacin for 3 days. Cytomegalovirus (CMV) infection was defined by the appearance of CMV antigen polymerase chain reaction in the blood. This measure was performed once a week.

### Statistics

Linear regression was used in univariate analysis to identify predictors of donor or recipient PCT concentrations. Predictors with a *P *value of less than 0.1 in univariate analysis were included in a multivariable linear regression model, with a stepwise variable selection method. Potential associations between graft dysfunction or overall complications and clinical or biological parameters were tested with univariate procedures, using Mann-Whitney tests for continuous variables and chi-square or Fisher exact tests for categorical variables. The evolution over time of recipient PCT concentration was studied with an analysis of variance for repeated measurements. The multiplicity associated with the comparisons between times was addressed using Scheffe adjustments. The alpha level was set at 0.05. All analyses were performed with the SAS software version 8.2 (SAS Institute Inc., Cary, NC, USA).

## Results

Sixty-seven patients (19 women and 48 men) were included. Thirty-eight were transplanted because of postviral hepatitis cirrhosis, 19 because of alcoholic cirrhosis, and 10 for other causes. The main characteristics of donors are reported in Table 1. Infection was confirmed in 4 cases by positive bloodstream culture (2 Gram-positive and 2 Gram-negative) and in 5 cases by positive bronchoalveolar lavage fluid sample (2 Gram-positive and 3 Gram-negative). In 3 cases, no microorganism was found but the patient was already treated with antibiotics and the clinical presentation (fever hypoxemia and hyperleukocytemia with chest radiologic abnormality) was strongly evocative of pneumonia. The main characteristics of recipients before OLT are reported in Table 2. None of them received catecholamines or had severe infection before OLT. Of the 67 patients, 12 presented hepatic dysfunction after OLT: 8 with pulmonary complications and 4 with renal complications (2 patients were dialysed after day 8). No patient presented PNF. Of the other 55 OLT patients without hepatic dysfunction, we observed 11 pulmonary complications and 3 renal complications. No patient required post-liver transplantation catecholamines. In 4 patients, a significant growth of quantitative cultures of distal bronchoalveolar lavage was demonstrated (cocci Gram-positive). No patient presented acute rejection before day 7. Two patients with initial hepatic dysfunction died at months 2 and 4, without hospital discharge, and one other patient died without initial hepatic dysfunction.

**Table 1:** Main donor characteristics

Main donor characteristics	Number or mean ± standard deviation
Age, years	48 ± 16
Gender, male/female	38/29
Epinephrine or norepinephrine dose, mg/hour	2.4 ± 2.7
Cardiac arrest, yes/no	10/57
General infection, yes/no	12/55
Days in the intensive care unit	3.6 ± 3.5
Heart retrieval, yes/no	36/31
Procalcitonin concentration, ng/mL	4.5 ± 14.6
Acute renal failure	0

**Table 2:** Main recipient characteristics

Main recipient characteristics	Number or mean ± standard deviation
Age, years	50 ± 11
Cold ischemia, minutes	485 ± 99
Warm ischemia, minutes	56 ± 18
Veno-venous bypass, yes/no	16/51
Very severe portal hypertension, yes/no	39/28
Low tolerance to liver vascular exclusion, yes/no	13/54
Operative transfusion, blood cell packs	6.4 ± 3.7
Alanine aminotransferase peak, UI/L	1,455 ± 1,527
Aspartate aminotransferase peak, UI/L	871 ± 981
Acute renal failure before transplantation	0

### Procalcitonin in recipients

**Figure 1:**
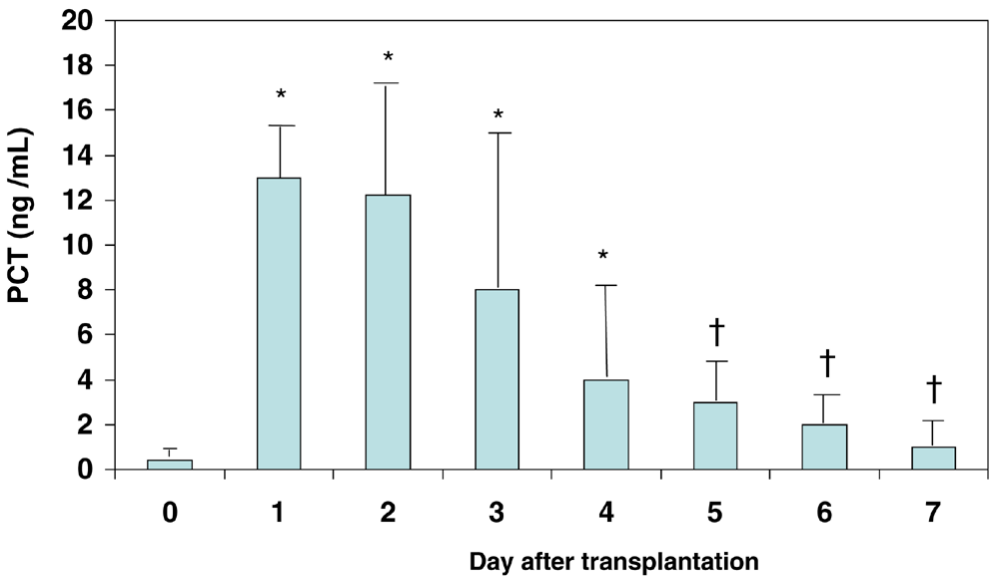
Time course of procalcitonine (PCT) in the recipient before liver transplantation and during the first week after liver transplantation. Results are expressed as mean ± standard deviation. *P < 0.05 (versus D0), ^†^*P *< 0.05 (versus D1).

PCT concentration was normal, less than 0.5 ng/mL, in 61 recipients before OLT (D0). Cause of transplantation did not influence PCT level in recipients before total hepatectomy: median 0.1 ng/mL (range 0.1 to 0.8) versus 0.1 ng/mL (range 0.1 to 2) versus 0.2 ng/mL (range 0.1 to 1.1) in cirrhosis from viral, alcoholic, and other causes, respectively. Peak PCT values were observed at D1 or D2. Then, PCT mean concentrations decreased from D2 to D7. Mean values are reported in Figure 1. A second increase in PCT was observed at D6 in five patients whose bacterium was isolated in bronchoalveolar lavage (without the need of mechanical ventilation). PCT concentration at D0 did not significantly differ in either type of recipient (with or without hepatic dysfunction): median 0.1 ng/mL (range 0.1 to 0.3) versus 0.1 ng/mL (range 0.1 to 3). PCT peak serum level (D1) was not significantly different in recipients with hepatic dysfunction versus no hepatic dysfunction: median 7.8 ng/mL (range 1.1 to 45) versus 7.3 ng/mL (range 0.6 to 85). PCT at D0 or D1 in recipients who would develop a complication was not different from that in recipients who would not. Parameters in multivariate analysis significantly associated with a peak in recipient PCT concentration (D1) were occurrence of CA in the 24 hours before retrieval in the donor and presence of infection in the donor (Table 3). The adjusted mean PCT concentration at D1 was 32.1 ng/mL (95% confidence interval [CI] 24.1 to 40.1) versus 16.3 ng/mL (95% CI 11.9 to 20.8) in patients with and without CA in the donor. The mean adjusted difference between the two groups was 15.8 ng/mL (95% CI 6.9 to 24.6). The adjusted mean PCT concentration at D1 was 32.9 ng/mL (95% CI 25.6 to 40.3) versus 15.5 ng/mL (95% CI 10.7 to 20.4) in patients with and without infection in the donor. The adjusted mean difference between the two groups was 17.4 ng/mL (95% CI 9.2 to 25.7).

**Table 3:** Multivariate analysis of predictive factors of peak concentration of procalcitonin in recipients

Variable	*P *univariate	*P *multivariate
Age of recipient	0.7	NS
Cold ischemia	0.97	NS
Warm ischemia	0.18	NS
Veno-venous bypass	0.34	NS
Liver vascular exclusion tolerance	0.01	NS
Transfusion of recipient	0.22	NS
Procalcitonin donor concentration	0.005	NS
Pre-liver transplantation procalcitonin concentration	0.37	NS
Severe portal hypertension	0.07	NS
Epinephrine or norepinephrine doses in donor	0.07	NS
Days in intensive care unit of donor	0.03	NS
Age of donor	0.5	NS
Heart retrieval	0.16	NS
Cardiac arrest in donor	<0.0001	0.001
Infection in donor	<0.0001	0.0039

NS, not significant.

### Procalcitonin in donor

PCT was normal in 38 donors. Median PCT concentrations were 0.8 ng/mL (range 0.1 to 8.7) in 49 patients I^-^/CA^-^, 16.5 ng/mL (range 0.2 to 91) in 6 patients I^-^/CA^+^, 1.8 ng/mL (range 0.7 to 4.7) in 8 patients I^+^/CA^-^, and 1.1 ng/mL (range 0.1 to 10.1) in 4 patients I^+^/CA^+^. The multivariate analysis did not show any association between donor PCT concentration and OLT hepatic dysfunction or overall complications. The parameters studied for this analysis were donor age, donor PCT level, cold ischemia, warm ischemia, operative transfusion, veno-venous bypass, a high dose of catecholamines in donors, CA and infection in donors, days in intensive care of donor before organ retrieval, and age of recipient. Donor age was the only parameter associated with hepatic dysfunction in univariate analysis (*P *= 0.03). In this model (adjusted with age), the median levels of PCT were 6.7 ng/mL (range 0.56 to 85) in recipients without hepatic dysfunction and 8.7 ng/mL (range 1.13 to 45) in recipients with hepatic dysfunction. Donor parameters significantly associated in multivariate analysis with donor concentration of PCT were a dose of epinephrine or norepinephrin administered before liver retrieval and occurrence of CA in the 24 hours prior to retrieval (Table 4).

**Table 4:** Multivariate analysis of predictive factors of peak concentration of procalcitonin in donors

Variable	*P *univariate	*P *multivariate
Days in intensive care unit of donor	0.52	
Age of donor	0.5	
Heart retrieval	0.16	
Infection in donor	0.75	
Cardiac arrest in donor	0.0001	0.003
Epinephrine or norepinephrine doses in donor	0.002	0.046

## Discussion

This study could not confirm the hypothesis that the donor PCT could be predictive of hepatic dysfunction or post-OLT complications in the recipient. Second, it showed that a peak in PCT in the recipient was associated with the clinical characteristics of donors but not with recipient characteristics and post-OLT complications. Third, our study confirmed the time course of PCT serum concentration in recipients after liver transplantation [5,6]. Conditions of sampling, timing after graft flushing, and the technique of the graft flushing were well standardized as Fazakas and colleagues [8] proposed in order to avoid any bias in intensity of PCT peak after reperfusion. Our results were compatible with other studies about the normal range of serum PCT levels [9-11] in cirrhotic patients without infection.

### Liver graft dysfunction and postoperative complications

Our results were not in agreement with those of Fazakas and colleagues [12], who found higher PCT peak levels in patients with postoperative complications. Precise assessment of the liver donor is essential because this is an important prognostic factor for outcome after OLT [13]. The decision to accept a donor liver is based on many variables, such as medical history, hemodynamic parameters, vasopressive support, laboratory parameters, liver echography, and (in selected cases) liver biopsy. The visual inspection is a subjective parameter to rule out major liver diseases such as cirrhosis or major steatosis. In contrast with heart transplantation, when elevated donor PCT did indicate early graft failure [14,15], we did not confirm these results, even after adjusted analysis. Postoperative hepatic dysfunction and complications are multifactorial processes that are probably too complex to be predicted by a sample marker such as donor PCT level or early post-OLT PCT level.

### Procalcitonin and infection

Our study did not find any association in donors between PCT concentration and infection, which is in contrast to the current concept [4]. To assess the diagnosis of infection, fever or white blood cell count is the most-used parameter, however unspecific it may be. Brain death, the associated adrenergic storm, and subsequent physiopathological changes make the diagnosis of infection difficult. Many multiple-organ donors require fluid resuscitation with plasma expanders and vasopressor therapy. The difficulty of assessing infection in donors and the lack of sepsis severity in some cases could explain the absence of an increase in PCT in donors in our study. Indeed, a few years ago, PCT was identified as a marker of inflammatory host responses which is particularly induced in severe bacterial infections and sepsis [3]. Not infection *per se *but infection associated with a severe systemic response or poor organ perfusion is thought to induce PCT release [16]. In the present study, PCT levels were elevated only in a minority of donors and no difference was found in donors with infection or not. An explanation could be that infection was not so severe as to induce PCT production in the donor. Maybe infection, even not severe infection, could induce modification in the liver, with a subsequent increase in PCT production in the recipient. It could be supposed that hepatic monocytes [17] exposed in the donor to infection (not severe) did not produce high levels of PCT in the donor but that a second exposure to another stimulus (like ischemia or reperfusion) would produce major amounts of PCT in the recipient. Moreover, the dramatic increase in PCT in some CA donors could have masked the effect of infection. Concerning post-OLT infection, the absence of severe sepsis in the first postoperative week, probably because of the broad-spectrum antibioprophyllaxis [18,19], makes this parameter difficult to analyze.

### Cardiac arrest and organ perfusion

Although some authors demonstrated toxicity of PCT [20], the physiopathology of increase in PCT is usually considered as the immune activation induced by the intestinal malperfusion in relation to cardiac dysfunction or CA. It has been speculated that PCT may be induced by endotoxin translocation and proinflammatory cytokines in these situations [21,22]. Even dramatic PCT increases in OKT3/ATG-treated patients could have been induced by increased enteral permeability with endotoxin translocation [23,24]. Many articles reported a PCT increase after cardiac surgery with extracorporeal circulation [25-27], especially in the presence of complications. In patients with heart stroke, hyperprocalcitoninemia was also reported [28], especially in patients with severe heart failure or in cases of resuscitation after CA [29,30]. Furthermore and probably because donor resuscitation has been improved, some authors demonstrated that liver grafts from CA donors functioned similarly to grafts from non-CA donors [31]. Our results are in agreement with these various reports: PCT peak is produced with higher intensity in donors with cardiac instability or with CA probably because of an inflammatory response induced by intestinal hypoperfusion. However, in our study, this production was not statistically associated with hepatic function: maybe because liver graft recovery is more sensitive to intrinsic liver quality than to cardiac events before retrieval in donors, we decided to retrieve the liver. Moreover, because of the little size of our seria, caution in the conclusion of non significant results of multivariate analysis is required.

## Conclusion

PCT level in the donor could not be considered as a good predictive marker of hepatic dysfunction or postoperative complication. PCT in the donor was associated with CA but not with infection. Post-OLT PCT peak is associated with infection and CA in donor but not in recipient parameters.

## Key messages

• Procalcitonin (PCT) level in the donor and early PCT peak in the recipient are not associated with postorthotopic liver transplantation hepatic dysfunction or other complications.

• PCT in the donor is associated with cardiac arrest but not with infection.

• High PCT peak levels in recipients are associated with infection and cardiac arrest in donors.

## Abbreviations

CA = cardiac arrest; CI = confidence interval; CMV = cytomegalovirus; I = infection; OLT = orthotopic liver transplantation; PCT = procalcitonin; PNF = primary nonfunction.

## Competing interests

The authors declare that they have no competing interests.

## Authors' contributions

DE conceived, designed, and carried out the study. SBA and MB helped to conceive and carry out the study and helped to perform laboratory analyses. MLT helped to conceive and carry out the study and performed statistical analyses. JMS helped to conceive and carry out the study. CV and SF helped to perform the literature search. MM and J-CV helped to compile the data for the study and helped to perform analyses. PC and LH helped to conceive the study. All authors contributed to the writing of the manuscript and approved of its final version.
